# Polymer Backbone Stabilized Methylammonium Lead Bromide Perovskite Nano Islands

**DOI:** 10.3390/nano13202750

**Published:** 2023-10-12

**Authors:** Chinna Bathula, Soniya Naik, Atanu Jana, Ramasubba Reddy Palem, Aditya Narayan Singh, Mohammad Rafe Hatshan, Suresh D. Mane, Hyun-Seok Kim

**Affiliations:** 1 Division of Electronics and Electrical Engineering, Dongguk University-Seoul, Seoul 04620, Republic of Korea; cdbathula@dongguk.edu; 2 Chemical and Materials Engineering Department, University of Alberta, Edmonton, AB T6G 2H5, Canada; soniya.d.naik1990@gmail.com; 3 Division of Physics and Semiconductor Science, Dongguk University, Seoul 04620, Republic of Korea; atanujanaic@gmail.com; 4 Department of Medical Biotechnology, Dongguk University, 32 Dongguk-ro, Ilsandong-gu, Goyang 10326, Republic of Korea; palemsubbareddy@gmail.com; 5 Department of Energy and Materials Engineering, Dongguk University-Seoul, Seoul 04620, Republic of Korea; aditya@dongguk.edu; 6 Department of Chemistry, College of Science, King Saud University, P.O. Box 2455, Riyadh 11451, Saudi Arabia; mhatshan@ksu.edu.sa; 7 D.Y. Patil Pratisthan’s College of Engineering, Salokhe Nagar, Kolhapur 416007, Maharashtra, India; mane.suresh@gmail.com

**Keywords:** nano islands, perovskites, stable, PDT, optical

## Abstract

Organic-inorganic hybrid perovskite materials continue to attract significant interest due to their optoelectronic application. However, the degradation phenomenon associated with hybrid structures remains a challenging aspect of commercialization. To overcome the stability issue, we have assembled the methylammonium lead bromide nano islands (MNIs) on the backbone of poly-3-dodecyl-thiophene (PDT) for the first time. The structural and morphological properties of the MNI-PDT composite were confirmed with the aid of X-ray diffraction (XRD) studies, Field emission scanning electron microscope (FESEM), and X-ray photoelectron spectroscopy (XPS). The optical properties, namely absorption studies, were carried out by ultraviolet-visible spectroscopy. The fluorescent behavior is determined by photoluminescence (PL) spectroscopy. The emission peak for the MNI-PDT was observed at 536 nm. The morphology studies supported by FESEM indicated that the nano islands are completely covered on the surface of the polymer backbone, making the hybrid (MNI-PDT) stable under environmental conditions for three months. The interfacial interaction strategy developed in the present work will provide a new approach for the stabilization of hybrids for a longer time duration.

## 1. Introduction

In the past few decades, the solar cell research community has witnessed a resurgence of interest in perovskites. Perovskites are potential candidates for use in various energy storage and conversion applications [1,2]. Research interests, particularly in metal halide perovskites [3], are motivated by their multifarious applications in energy storage and conversion applications, such as solar cells [4], light-emitting diodes [5], X-ray imagers [6], photoelectrochemical applications [7,8], lasers [9], and so on. These halide perovskites are represented as ABX3, where “A” corresponds to Cs^+^ or methylammonium (CH_3_NH_3_^+^, MA^+^), or formamidinium (NH_2_ = CHNH_2_^+^, FA^+^), “B” could be divalent metals such as Pb^2+^ or Sn^2+^, and “X” is anion in VII A group (halide) in periodic board, specifically Br, I, or Cl [10]. Despite their wider applicability, the halide perovskites are commercially plagued due to their structural instabilities. These structural uncertainties are due to the unstable ionic connection concerning cationic and anionic segments through their establishment. Thus, protecting these ionic nanostructures against moisture, heat, and light is essential for their wider implementation in practical applications.

Methylammonium lead bromide (MAPbBr_3_) is known to degrade in the presence of moisture, heat, light, and other degrading factors. In literature, it is reported that its stability is somewhat better than MAPbI_3_, due to stronger Pb-Br bonds [11,12]. The strong bond between Pb-Br increases the halide ion defect formation energy, thereby suppressing the degradation due to ion migrations. Cation migration of bulky MA^+^ could also be restricted by steric hindrance, due to smaller lattice constants of MAPbBr_3_ and having a stronger hydrogen bonding to the [PbBr_6_]^4−^ octahedra. Despite this, migration has been the root cause of degradation for this compound [13]. Thus, substantial efforts are needed to stabilize this material to extract maximum benefits.

However, stabilization techniques advocating the partial replacements of A-site or halide sites are reported by several research groups [14]. Quite often, Cs^+^ is partially substituted in the lattice structure, which results in improved thermal stability. Nonetheless, it brings lattice reduction in the virgin MAPbBr_3_ structure owed ionic radii disparity between MA+ and Cs+ ions and results in octahedral tilting of the parent crystal [15]. Furthermore, it has been reported that varied cations and halides show an added advantage over predictable halide perovskites in terms of its steadiness, tunable band gap, enhanced performances, increased charge carrier transport, and reduced hysteresis [16]. Graphene nanosheets (NSs) bearing different chemical groups have also been proposed as an effective tool to control, tune, and stabilize perovskite nanocrystals (NCs) for their practical applications. Using graphene as a scaffold to encapsulate perovskites has the advantage of exceptional charge transport possessions; it has also been extensively used as a charge transport layer in perovskites-based solar cells [17,18]. Although such techniques have proved successful on several occasions, oxygen containing functionalities significantly alters the growth process, and obtained perovskites are rich in oxygen defects [19]. Surface functionalities such as phenylamine, iodine, and embedded nitrogen atoms or nitrogen/sulfur co-doping strategies [20,21,22] also significantly alter the growth process and are thus unsuitable for certain applications.

Furthermore, stabilizing 3D hybrid halide perovskites is another daunting task. Recently, entropic stabilization effect has been reported in a new class of highly defective 3D halide perovskites with high crystallinity, which the author has termed “hollow” perovskites [23]. The term hollow perovskite emanates from extensive Pb and X vacancies created on the occupancies/insertions of the ethylenediammonium (en) cation. The hollow motif allows a tailor-made profile for various optoelectronic applications of this material. The calculated formation energies and enthalpy suggests that bromide perovskites have a greater propensity toward stability compared to their iodide counterparts. The stability difference is not far to seek, as it stems from the 3D cage size differences in bromide and iodide structures. The enhanced stability of bromide-based perovskite is due to configurational entropy, which arises due to randomly distributed cations and anions vacancies in the perovskite. Furthermore, the increased defects due to incorporations restrict the halide migrations and stabilize the crystal structure. Another effort to stabilize MAPbBr_3_ was reported, which consists of one-step, is solvent free, and follows a green route [24]. This strategy uses metal-organic frameworks (MOFs) of perovskite [MA-M(HCOO)_3_], where M can be Mn/Co. In this process, the perovskite MOF serves as both a template and a source of MA cations for the growth and stabilization of hybrid perovskite. The composite material is synthesized mechanochemically by grinding the perovskite MOF with PbBr_2_, eliminating the need for external reagents. The resulting MAPbBr_3_@MA-Mn(HCOO)_3_ composite exhibits remarkable chemical stability in various solvents. Additionally, it demonstrates excellent processability and can be used as an electrode material, displaying photoelectrochemical activity when exposed to light. This innovative approach offers a sustainable and efficient means of fabricating stable perovskite-based materials with potential applications in optoelectronics and beyond. Likewise, stabilizing by polystyrene (PS) fiber membrane using an electrospinning technique has also been reported [25]. The obtained composite fiber MAPbBr_3_@PS showed excellent properties particularly 70% fluorescence intensity, even after being soaked for 30 days in water and maintained ~90% after UV light irradiation for 100 h. This remarkable stability is attributed to the formation of hydrogen bonds between MAPbBr_3_ and PS fibers. Halide perovskites can also be stabilized by growing them in in-situ conditions. Recently, it was reported that during in-situ growth of perovskite within a polyvinylidene fluoride (PVDF) polymer matrix, could significantly improve the hydrothermal stability [26]. The enhanced performances are due to the introduced tetradecyl phosphonic acid (TDPA) ligands, which form strong-covalent P-O-Pb bonds with the perovskite surface. This enhanced binding not only effectively passivates surface defects on the perovskites, but also minimizes ligand desorption from the perovskite surfaces at high temperatures. Consequently, the photoluminescence quantum yield (PL QY) of the MAPbBr_3_-grown at PVDF film remains almost unchanged after exposure to air at 333 K for 1800 h. Even at elevated temperatures ranging from 293 to 353 K, the PL intensity remains at 88.6% of the initial value, a significant improvement compared to perovskites without the TDPA ligand (42.4%).

In another study, to stabilize MAPbBr_3_ and to produce a large scale could be achieved by emulsion electrospinning technique to result in MAPbBr_3_@polymer hybrid films [27]. In this process, polymethyl methacrylate (PMMA) solidifies into an outer shell layer during electrospinning. Simultaneously, emulsion drops containing PVDF and perovskite precursor are pushed inward and transform into perovskite nanocrystals covered by PVDF. The smooth surface of PMMA enhances light transport, while the water-resistant PVDF layer provides protection against moisture. The resulting fibers embedded with methylammonium lead bromide perovskite exhibit strong light emission even after being stored in a humid ambient environment (with relative humidity > 60%) or submerged in water. The study demonstrates amplified spontaneous emissions from the network of these fibers and waveguide lasing from individual chopped fibers. This approach offers a promising path toward highly stable perovskite lasers with practical applications. Stabilization of perovskites could also be achieved by MOFs. A zeolitic imidazolate framework (ZIF-8), using pore encapsulated solvent-directed (PSD) approach, could be used to stabilize perovskites [28]. This perovskite maintained its stability even after being dipped into wide-ranging polar solvents, such as boiling water, and after prolonged exposure to UV irradiations. This immense stabilization stems from the protection layer from the walls of MOFs, which restricts any degradation to attack the perovskite, thereby ensuring long-term stability.

To improve the stability of perovskites, all the strategies could be grouped into three categories: (1) stronger binding ligands modifying the OLA/OA caped perovskites, (2) encapsulation with polymers or hydrophobic polymers, and (3) composite heterostructures with different perovskites phases [29]. Although several of these strategies, such as the use of inorganic oxides (SiO_2_, AlO_x_, and so on), physically isolate perovskites, thereby ensuring stability, they result in significantly reduced solution processability of the encapsulated materials [30,31]. Thiophene-based polymers such as poly-3-dodecyl thiophene (PDT) with regioregularity are the most promising semicrystalline polymers owing to their extraordinary charge carrier mobility and respectable solubility in organic solvents. PDT nanostructures must be created with various scales and excellent morphologies to produce high-performance organic-inorganic hybrid materials for their applications in electronic designs. For stabilizing the perovskite, various block copolymers consisting of PDT are examined, due to their self-assembly ability to form periodic nanostructures. However, most poly(3-alkylthiophene) containing block copolymers usually exhibit only fibril morphology due to strong rod/rod interaction inside the molecule. Herein, we report the stabilization of perovskites by a polymeric backbone to significantly enhance its structural stability. The structural and morphological properties of the MNI-PDT composite were confirmed by the aid of X-ray diffraction (XRD) studies, Field emission scanning electron microscope (FESEM), X-ray photoelectron spectroscopy (XPS). The optical properties, namely absorption studies, were carried out by ultraviolet-visible spectroscopy. The fluorescent behavior is determined by photoluminescence (PL) spectroscopy. The assembly of MNI on PDT polymer improves the stability of MNI for application in optoelectronics. The long chain alkyl cations of MNI and sulfur of PDT polymer helped preserve their stability for over three months. The synthesized MNI-PDT displayed a peak of emission 536 nm. Furthermore, the substantial MNI-PDT indicated intensity upon storing in ambient conditions. The reliability of MNI-PDT was validated by operating XRD on an MNI-PDT that was stored for three months. The PL measurement of the assembled sample over the longer period of time further demonstrates the stability of the material after storage in ambient conditions. This method provides new directions for stabling nanostructure through polymer and perovskite interactions.

## 2. Materials and Methods

### 2.1. Materials

The solvents Dimethyl formamide (DMF, HCON(CH_3_)_2_, 99.8%) and Toluene (C_6_H_5_CH_3_, 99.8%) of the highest grade were precured from Sigma-Aldrich, Seoul, Republic of Korea, and used as received without further purification. The precursors for perovskite MNI, the C_8_H_17_NH_3_Br and CH_3_NH_3_Br, were prepared by reported literature method [32,33].

### 2.2. Synthesis of Methylammonium Bromide (CH_3_NH_3_Br) and Octylammonium Bromide (C_8_H_17_NH_3_Br)

The HBr (83.33 mL, 0.5 mol) was added dropwise to methylamine (42.86 mL, 0.5 mol) or n-octylamine (82.74 mL, 0.5 mol) dissolved in 50 mL ethanol were conducted at 0 °C with magnetic stirring for two h. The excess ethanol evaporated using a rotary evaporator while the water bath temperature was maintained at 60 °C, yielding a white solid product. The product was again dissolved in ethanol for a second time and added to the precipitate. The solid was filtered out and vacuum-dried at 60 °C in an oven for six h for future use.

### 2.3. Synthesis of Pure MAPbBr_3_ (MNI) NCs

In a 30 mL glass vial, PbBr_2_ (146.8 mg, 0.4 mmol), CH_3_NH_3_Br (17.6 mg, 0.16 mmol), and C_8_H_17_NH_3_Br (50.4 mg, 0.24 mmol) were dissolved in 4 mL N, N-dimethylformamide (DMF) and kept at 80 °C for 10 min with constant stirring. Then, as a precipitating solvent, 10 mL toluene was added. The precipitate was centrifuged for five min at 5000 rpm. The product was dried at 60 °C in an oven for six h. 

### 2.4. Preparation of MNI-PDT

The starting materials PbBr_2_ (73.4 mg, 0.2 mmol), CH_3_NH_3_Br (8.8 mg, 0.08 mmol), and C_8_H_17_NH_3_Br (25.2 mg, 0.12 mmol) were added to 2 mL DMF in a 10 mL vial and ultrasonicated to make it clear and then added to 50 mg PDT polymer dispersed in 5 mL DMF in a 20 mL vial. The reaction combination is warmed to 80 °C for 15 min, followed by the addition of toluene to give finely dispersed yellow powder. The product was centrifuged at 5000 rpm for five min, dried at 60 °C for six h, and utilized for characterization and optical studies. Thus, prepared MNI-PDT was stored in ambient conditions.

### 2.5. Characterization Methods

Surface characteristics of MNI-PDT were determined using X-ray photoelectron spectroscopy (XPS) by means of the K-alpha instrument (Thermo-Fisher, Waltham, MA, USA). The morphological properties of MNI-PDT were analyzed by Field emission scanning electron microscopy (FESEM) using the MIRA II KMH-TESCAN instrument. Energy-dispersive X-ray spectroscopy (EDS, X-act6, OXFORD) analysis was performed to determine elemental composition. The phase analysis to understand the level pureness of the sample was considered by XRD with Bragg’s diffraction 2θ (10 to 50°) with a 2°/minute scan rate by X-ray diffraction (Rigaku-D/MAX2500V/PC, Cedar Park, TX, USA). The absorption parameters were measured with the aid of a UV-VIS spectrophotometer (JASCO V-770, Oklahoma City, OK, USA). The photoluminescence characteristics were determined by a Cary Eclipse fluorometer (Varian, Palo Alto, CA, USA). Functional groups present in composite were studied by Fourier transmission Infrared spectroscopy (FTIR) by Bruker Optik GmbH, Ettlingen, Germany (FTIR, Vertex-70V/Hyperion 3000).

## 3. Results and Discussion

**Figure 1:**
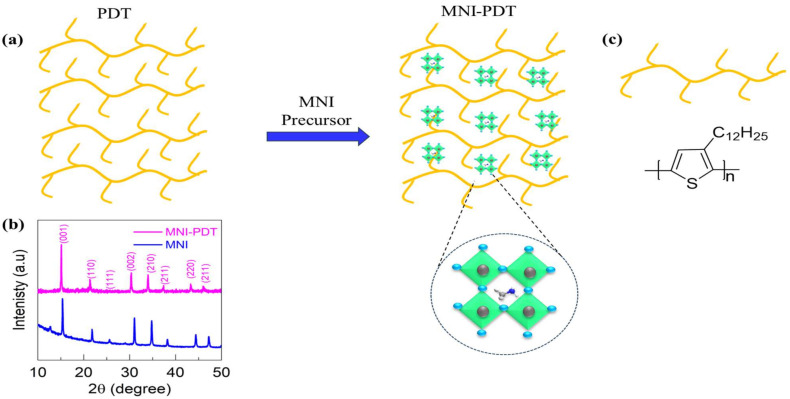
(**a**) Schematic of MNI-PDT composite formation, (**b**) XRD spectra for MNI and MNI-PDT composites, (**c**) Chemical structure of PDT polymer and a schematic diagram of its molecular skeleton.

The ligand aided the precipitation process to assemble MNI-PDT which is achieved via a two-stage protocol. In the initial stage, the precursors of MNI were dissolved in DMF in a glass vial and added to a vial containing PDT polymer dispersed in DMF in a glass vial. Finally, during the second step the toluene is added into the reaction tube to obtain MNI-PDT powder as a dispersion. The product was centrifuged at 5000 rpm for five min and dried at 60 °C for six h. A representation of the synthetic protocol for the preparation of MNI-PDT is illustrated in Figure 1. Among the numerous extrinsic influences that commence the degradations in perovskite, moisture/humidity is crucial. Furthermore, the organic cations in perovskites are greatly hygroscopic in character and arise weak hydrogen bonds in the manifestation of moisture/humidity. Several classes projected that reactions within the perovskite under the influence of moisture are both changeable and permanent. Indeed, the degradation of perovskites under the influence of moisture is the first degradation route, and thus it has been inspected systematically. The reactions are reversible to a limit with inadequate access to moisture. The excess entry of moisture in perovskites results in the formation of a transition between mono- and di-hydrate structured architecture. Although, under dry air, these reactions can be converted back to their original state in a limited time frame. In the absence of dry air, the presence of hydrogen bonds in the hydrated species weakens the bond between cations and the octahedral PbI_6_, and this reaction is further accelerated under light/heat/oxygen, leading to deprotonation of cations and, finally, degradation of perovskites. The well-decorated assembly of polymers and perovskites is a promising strategy to overcome degradation and stabilize the perovskites.

X-ray diffraction (XRD) is a universal representation practice for nanoscale resources. Analysis of a sample by XRD grants important figures that correspond to numerous microscopic and spectroscopic techniques, such as phase identification, sample purity, crystallite size, and, in some cases, morphology. As a bulk technique, the evidence it provides can be connected with microscopy data to test if microscopic statements on a small number of particles are characteristic of the greater part of the sample. The organizational growth of the MNI-PDT and its phase purity are elucidated by X-ray diffraction (XRD). The XRD compiled MNI-PDT, reveals the level of crystal structure is revealed in Figure 1b. The MNI on PDT generated XRD peaks at 15.15°, 21.40°, 25.57°, 30.32°, 34.09°, 37.42°, 43.34°, 46.01° for planes (001), (110), (111), (002), (210), (211), (220), and (211), confirming the manifestation of MAPbBr_3_. In addition, there are no significant signals observed for the polymer due to its amorphous nature. However, the XPS and EDX manifested the presence of sulfur from the PDT polymer. The clear and apparent XRD heights signify the growth of MNI. The cations of octylammonium were additionally unearthed to promote the firmness of the nanostructure. The FTIR spectra are shown in Figure S1 where the characteristic signals of polymer and perovskite are clearly in the MNI-PDT network.

**Figure 2:**
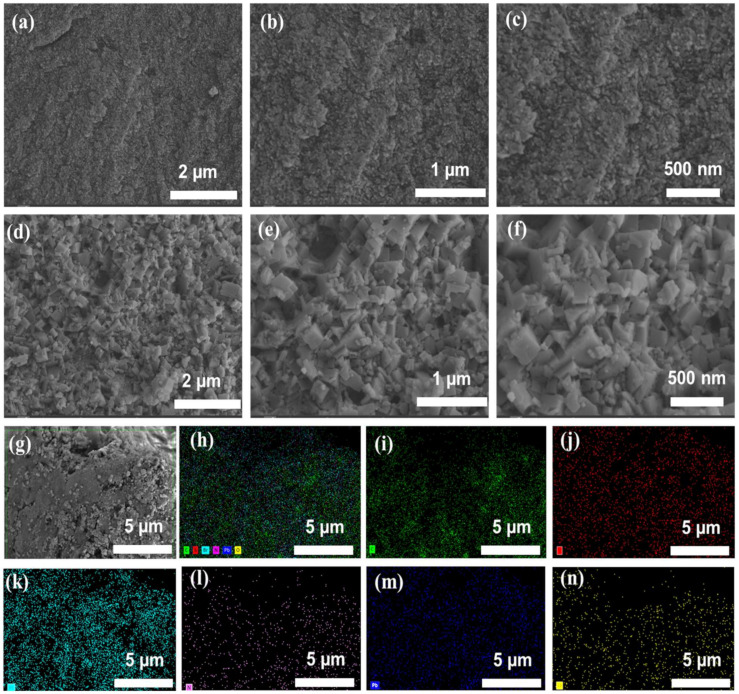
SEM images at different magnifications. (**a**–**c**) PDT, (**d**–**f**) MNI-PDT, and (**g**–**n**) EDS mapping showing the elements C, S, Br, N, Pb and O.

Field emission scanning electron microscopy (FESEM) utilizes topographical and elemental information at magnifications with fundamentally infinite depth of field. It can also examine smaller-area contamination spots at electron-accelerating voltages compatible with energy dispersive spectroscopy (EDS). To understand and reveal the surface morphology of perovskite nano islands on PDTs, field effect scanning electronic microscopic images were obtained, as shown in Figure 2. Images of PDTs are shown in Figure 2a–c, where the fibers of polymers are visible, whereas nano islands of perovskite are decorated on the PDT polymer as shown in Figure 2d–f. These nano islands can serve as a stabilizing agent for perovskite material. These islands can encapsulate and protect the perovskite, preventing direct exposure to environmentally degrading factors (moisture, heat, light, and other degrading factors). Not only do these nano islands assist in protecting the perovskite fragile structure, but also offer a better and uniform surface coverage leading to improved light absorption and charge transport properties. These islands can also reduce charge recombination at the perovskite/polymer interface leading to improved overall device performances (refer-In-situ growth of low-dimensional perovskite-based insular nanocrystals for highly efficient light emitting diodes). To further show the elemental distribution, EDS mapping is obtained. Figure 2g–n) shows a uniform distribution of elements C, S, Br, N, Pb, and O in MNI-PDT polymer indicating that polymer stabilization restricts the agglomeration and which in turn assists in long-term stability. 

**Figure 3:**
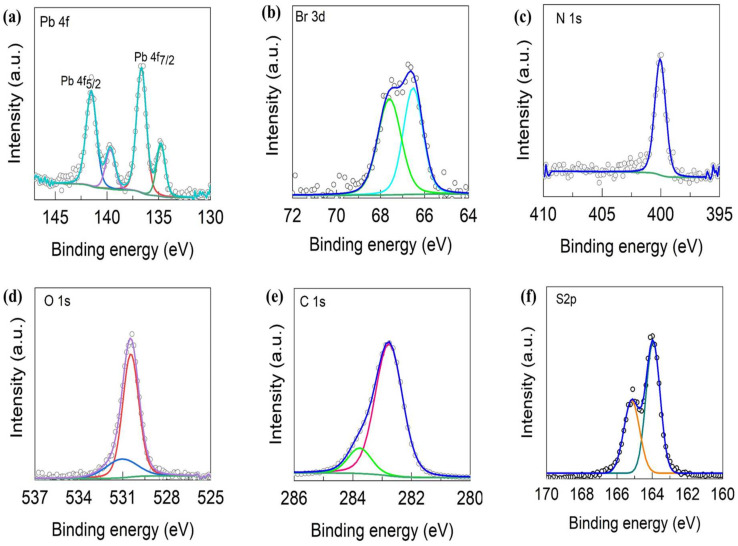
XPS spectra of MNI-PDT (**a**) Pb 4f, (**b**) Br 3d (**c**) N 1s, (**d**) O 1s, (**e**) C 1s, (**f**) S2p.

X-ray photoelectron spectroscopy (XPS) has become one of the most widely used surface analysis techniques. XPS can measure elemental composition as well as the chemical and electronic states of atoms in a material. XPS is used to support research on surface-mediated processes such as sorption, catalysis, redox, dissolution/precipitation, corrosion, and evaporation/deposition-type reactions. The chemical organization and its states of unique constituents in the MNI-PDT fabrication are resolved by X-ray photoelectron spectroscopy (XPS), as demonstrated in Figure 3. The XPS spectra are illustrated by Voigt curve fitting, following Shirley background elimination to designate the twin peaks of Pb 4f_7/2_ and Pb 4f_5/2_. Four peaks were obtained in the Pb 4f spectra, which reveals that Pb is represented in unique chemical environments. The peaks at 136.66 eV and 141.56 eV are assigned to Pb 4f_7/2_ and Pb 4f_5/2_ [34]. The spin-orbit split between the Pb Peaks is 4.9 [35]. The small peaks at 134.8 eV and 139.78 eV are designated to Pb^0^. This indicates that PDT potentially coordinates with Pb(0) through sulfur atoms and donates electron density, a phenomenon reminiscent of donor-π-acceptor (D-π-A) molecules [36]. The band at 67.49 eV is assigned to Br 3d. The signal at 400.1 eV is assigned to the N 1s [37] present in the perovskite skeleton. The O1s band is observed at 530.48 eV [38]. The carbon peak C 1s appeared at 283.18 eV [39]. The high-resolution spectrum of S2p shows two peaks at 164.7, which arise from spin-orbit coupling of the C-S bond in thiophene [40]. The survey spectra of MNI-PDT are illustrated in Figure S2. From the XPS it is concluded that MNI-PDT contains Pb 4f, Br 3d, N 1s, O 1s, C 1s, and S2p components in an assembled architecture. Hence, high-resolution XPS implies that MNI is efficiently shared with PDT to establish MNI-PDT. The analytical data obtained from SEM-EDS, XPS, and XRD confirms the successful establishment of MNI-PDT.

**Figure 4:**
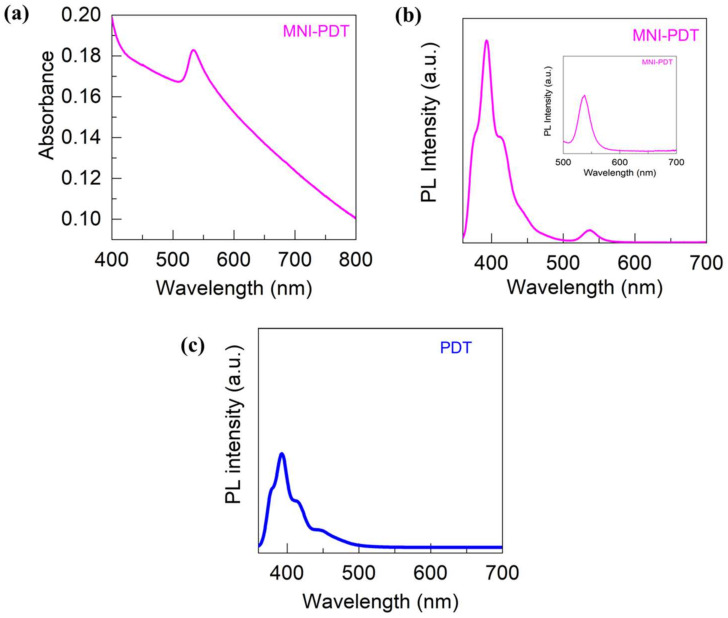
(**a**) Absorption spectrum of MNI-PDT, (**b**) PL spectrum of MNI-PDT (Inset-Magnified version), (**c**) PL spectrum of PDT.

**Figure 5:**
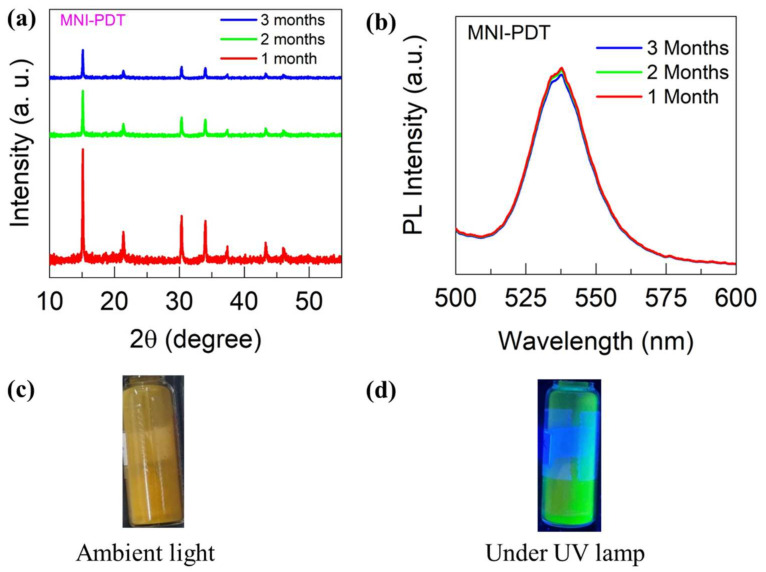
(**a**) XRD spectra for MNI-PDT after storing for 1, 2 and 3 months, (**b**) PL spectra for MNI-PDT after storing for 1, 2 and 3 months, (**c**) MNI-PDT at ambient light, (**d**) MNI-PDT under UV.

The opto-electronic parameters for MNI-PDT were established via PL, and the resulting data is illustrated in Figure 4. The absorption band at 533 nm for MNI-PDT (Figure 4a) corresponds to characteristics of MAPbBr_3_ [31]. The MNI-PDT gives a signal at 536 nm in PL, as confirmed in Figure 4b,c. This peak is slightly red-shifted from the absorption peak, which is common for perovskites. The PL spectrum also shows a signal at 392 nm, which is mainly due to the presence of polymers, as illustrated in Figure 4c. To determine the stability, MNI-PDT was stored at RT for three months, and XRD was carried out to identify substantial shifts in architecture of the fabricated material. The assumptions verify that the MNI-PDT reclaimed the similar pureness after three months (Figure 5a). Overall, the opto-electronic parameters of MNI-PDT are consistent with its composition and structure. The perovskite component gives MNI-PDT a strong absorption at 533 nm and a PL emission at 536 nm. The polymer component contributes a PL emission at 392 nm. MNI-PDT is also stable under ambient storage at room temperature for three months, as illustrated by PL (Figure 5b). The pictorial description of MNI-PDT under ambient light and UV are presented in Figure 5c,d. The fluorescent behavior of the sample is observed even in solid state.

The XRD signal intensity of our as-synthesized MNI-PDT sample decreased over time. While polymer encapsulation can significantly improve the stability of halide perovskite materials, it is important to note that it may not eliminate all degradation mechanisms. Over time, especially under prolonged exposure to environmental conditions, the polymer-encapsulated perovskite material may still experience some degree of degradation. Factors such as temperature, humidity, and the choice of encapsulation material can influence the long-term stability of the material. The sulfur atom in poly-3-dodecyl thiophene (P3DDT) plays a crucial role in binding and stabilizing halide perovskites by forming strong coordination bonds with metal cations, typically lead (Pb) in the perovskite structure [41]. This interaction occurs between the lone pairs of electrons on the sulfur atom and the positively charged metal ions, creating stable metal-sulfur (M-S) bonds. These bonds help anchor the P3DDT polymer to the perovskite surface, effectively overcoming the perovskite’s surface defects which are vulnerable to moisture and ion migration; this enhances the overall stability of the perovskite. The dodecyl side chains of P3DDT can also form hydrophobic interactions with the organic cations in the perovskite structure, creating a protective barrier against moisture and preventing water-induced degradation. The conducting polymer, such as poly-3-dodecyl thiophene, offers higher sharpness to the emission profile of the perovskite crystals and improves the purity of the emission color only through polymer perovskite interactions.

## 4. Conclusions

In summary, the NCs of perovskite were effectively draped on PDT to increase stability. The resulting MNI-PDT presented stability and moisture endurance. The morphological studies clearly indicated the incorporation of perovskite nano islands on poly-3-dodecyl thiophene in a well assembled architecture. Furthermore, our stability testing, involving a 3-month storage period at room temperature, was reassuring as XRD results confirmed that MNI-PDT maintained its purity and architectural integrity throughout this period. This long-term stability is ascribed to long-chain alkyl cations of MNI and sulfur of PDT polymers, which protects the perovskites against environmental degradation. Long-term stability is further supported by the formation of a uniform nano island, which acts as a barrier to environmentally degrading factors. The synthesized MNI-PDT displayed an emission peak of 536 nm. Furthermore, the substantial MNI-PDT indicated intensity upon storing in ambient circumstances. These findings demonstrate the potential of MNI-PDT as a stable and promising material for various opto-electronic applications. Further research can explore the practical implications of these properties in the development of efficient and reliable devices.

## Data Availability

Data available upon request.

## Supplementary Materials

The following supporting information can be downloaded at: https://www.mdpi.com/article/10.3390/nano13202750/s1, Figure S1: FTIR spectra for PDT, MNI-PDT and MNI.; Figure S2: XPS survey spectra for MNI-PDT.

## References

[1] Khenkin M.V., Katz E.A., Abate A., Bardizza G., Berry J.J., Brabec C., Brunetti F., Bulović V., Burlingame Q., Di Carlo A. (2020). Consensus statement for stability assessment and reporting for perovskite photovoltaics based on ISOS procedures. Nat. Energy.

[2] Singh A.N., Hajibabaei A., Diorizky M.H., Ba Q., Nam K.-W. (2023). Remarkably Enhanced Lattice Oxygen Participation in Perovskites to Boost Oxygen Evolution Reaction. Nanomaterials.

[3] Tsai H., Nie W., Blancon J.-C., Stoumpos C.C., Asadpour R., Harutyunyan B., Neukirch A.J., Verduzco R., Crochet J.J., Tretiak S. (2016). High-efficiency two-dimensional Ruddlesden–Popper perovskite solar cells. Nature.

[4] Jeong J., Kim M., Seo J., Lu H., Ahlawat P., Mishra A., Yang Y., Hope M.A., Eickemeyer F.T., Kim M. (2021). Pseudo-halide anion engineering for α-FAPbI3 perovskite solar cells. Nature.

[5] Tian Y., Zhou C., Worku M., Wang X., Ling Y., Gao H., Zhou Y., Miao Y., Guan J., Ma B. (2018). Highly efficient spectrally stable red perovskite light-emitting diodes. Adv. Mater..

[6] Jana A., Cho S., Patil S.A., Meena A., Jo Y., Sree V.G., Park Y., Kim H., Im H., Taylor R.A. (2022). Perovskite: Scintillators, direct detectors, and X-ray imagers. Mater. Today.

[7] Fehr A.M., Agrawal A., Mandani F., Conrad C.L., Jiang Q., Park S.Y., Alley O., Li B., Sidhik S., Metcalf I. (2023). Integrated halide perovskite photoelectrochemical cells with solar-driven water-splitting efficiency of 20.8%. Nat. Commun..

[8] Tiwari J.N., Singh A.N., Sultan S., Kim K.S. (2020). Recent Advancement of p- and d-Block Elements, Single Atoms, and Graphene-Based Photoelectrochemical Electrodes for Water Splitting. Adv. Energy Mater..

[9] Mizuno H., Nishimura T., Mekata Y., Kurahashi N., Odani M., Nguyen V.-C., Inada Y., Yamao T., Sasaki F., Yanagi H. (2021). Distributed feedback laser with methylammonium lead bromide embedded in channel-type waveguides. Jpn. J. Appl. Phys..

[10] Singh A.N., Kajal S., Kim J., Jana A., Kim J.Y., Kim K.S. (2020). Interface Engineering Driven Stabilization of Halide Perovskites against Moisture, Heat, and Light for Optoelectronic Applications. Adv. Energy Mater..

[11] Yoon S.J., Kuno M., Kamat P.V. (2017). Shift happens. How halide ion defects influence photoinduced segregation in mixed halide perovskites. ACS Energy Lett..

[12] McGovern L., Futscher M.H., Muscarella L.A., Ehrler B. (2020). Understanding the Stability of MAPbBr3 versus MAPbI3: Suppression of Methylammonium Migration and Reduction of Halide Migration. J. Phys. Chem. Lett..

[13] Nandal V., Nair P.R. (2017). Predictive modeling of ion migration induced degradation in perovskite solar cells. ACS Nano.

[14] Premkumar S., Kundu K., Umapathy S. (2019). Impact of cesium in methylammonium lead bromide perovskites: Insights into the microstructures, stability and photophysical properties. Nanoscale.

[15] Lin H.-C., Chen L.-Y., Lin T.-H. (2021). Improving hysteresis of room-temperature air-quenching MAPbI3-xClx solar cells by using mixed-lead halide precursor. Mater. Chem. Phys..

[16] Zhu Y., Jia S., Zheng J., Lin Y., Wu Y., Wang J. (2018). Facile synthesis of nitrogen-doped graphene frameworks for enhanced performance of hole transport material-free perovskite solar cells. J. Mat. Chem. C.

[17] Stergiou A., Sideri I.K., Kafetzi M., Ioannou A., Arenal R., Mousdis G., Pispas S., Tagmatarchis N. (2022). Methylammonium lead bromide perovskite nano-crystals grown in a poly [styrene-co-(2-(dimethylamino) ethyl methacrylate)] matrix immobilized on exfoliated graphene nano-sheets. Nanomaterials.

[18] Acik M., Park I.K., Koritala R.E., Lee G., Rosenberg R.A. (2018). Oxygen-induced defects at the lead halide perovskite/graphene oxide interfaces. J. Mater. Chem. A.

[19] Zhou Q., Tang S., Yuan G., Zhu W., Huang Y., Li S., Lin M. (2022). Tailored graphene quantum dots to passivate defects and accelerate charge extraction for all-inorganic CsPbIBr2 perovskite solar cells. J. Alloys Compd..

[20] Fu F., Pisoni S., Jeangros Q., Sastre-Pellicer J., Kawecki M., Paracchino A., Moser T., Werner J., Andres C., Duchêne L. (2019). I 2 vapor-induced degradation of formamidinium lead iodide based perovskite solar cells under heat–light soaking conditions. Energy Environ. Sci..

[21] Zhang Q., Zhou Y., Wei Y., Tai M., Nan H., Gu Y., Han J., Yin X., Li J., Lin H. (2020). Improved phase stability of γ-CsPbI 3 perovskite nanocrystals using the interface effect using iodine modified graphene oxide. J. Mat. Chem. C.

[22] Noel N.K., Abate A., Stranks S.D., Parrott E.S., Burlakov V.M., Goriely A., Snaith H.J. (2014). Enhanced Photoluminescence and Solar Cell Performance via Lewis Base Passivation of Organic–Inorganic Lead Halide Perovskites. ACS Nano.

[23] Jayanthi K., Spanopoulos I., Zibouche N., Voskanyan A.A., Vasileiadou E.S., Islam M.S., Navrotsky A., Kanatzidis M.G. (2022). Entropy Stabilization Effects and Ion Migration in 3D “Hollow” Halide Perovskites. J. Am. Chem. Soc..

[24] Rambabu D., Bhattacharyya S., Singh T., ML C., Maji T.K. (2020). Stabilization of MAPbBr3 Perovskite Quantum Dots on Perovskite MOFs by a One-Step Mechanochemical Synthesis. Inorg. Chem..

[25] Bi H., Liu F., Wang M., Mao Z., Zhai Y., Li W., Wang S., Zhang M. (2021). Construction of ultra-stable perovskite–polymer fibre membranes by electrospinning technology and its application to light-emitting diodes. Polym. Int..

[26] Fu H., Wang K., Wu H., Bowen C.R., Fang Z., Yan Z., Jiang S., Ou D., Yang Y., Zheng J. (2023). Enhanced Hygrothermal Stability of In-Situ-Grown MAPbBr3 Nanocrystals in Polymer with Suppressed Desorption of Ligands. Inorg. Chem..

[27] Wang Z., He H., Liu S., Wang H., Zeng Q., Liu Z., Xiong Q., Fan H.J. (2020). Air Stable Organic–Inorganic Perovskite Nanocrystals@Polymer Nanofibers and Waveguide Lasing. Small.

[28] Mollick S., Mandal T.N., Jana A., Fajal S., Desai A.V., Ghosh S.K. (2019). Ultrastable Luminescent Hybrid Bromide Perovskite@MOF Nanocomposites for the Degradation of Organic Pollutants in Water. ACS Appl. Nano Mater..

[29] Jiang G., Erdem O., Hübner R., Georgi M., Wei W., Fan X., Wang J., Demir H.V., Gaponik N. (2021). Mechanosynthesis of polymer-stabilized lead bromide perovskites: Insight into the formation and phase conversion of nanoparticles. Nano Res..

[30] Bathula C., Jana A., Opoku H., Youi H.-K., Ghfar A.A., Ahmed A.T.A., Kim H.-S. (2021). Interfacial engineering of quasi-2-D formamidinium lead iodide nanosheets for perovskite solar cell by mechanochemical approach. Surf. Interfaces.

[31] Bathula C., Jana A., Park Y., Kadam A., Sree V., Ansar S., Kim H.-S., Kim H. (2022). Facile synthesis and optical study of organic-inorganic lead bromide perovskite-clay (kaolinite, montmorillonite, and halloysite) composites. Surf. Interfaces.

[32] Mittal M., Jana A., Sarkar S., Mahadevan P., Sapra S. (2016). Size of the Organic Cation Tunes the Band Gap of Colloidal Organolead Bromide Perovskite Nanocrystals. J. Phys. Chem. Lett..

[33] Jana A., Mittal M., Singla A., Sapra S. (2017). Solvent-free, mechanochemical syntheses of bulk trihalide perovskites and their nanoparticles. Chem. Commun..

[34] Jana A., Ba Q., Kim A.N.K. (2019). Formation of a photoactive quasi-2D formamidinium lead iodide perovskite in water. J. Mater. Chem. A.

[35] Morgan W.E., Van Wazer J.R. (1973). Binding energy shifts in the x-ray photoelectron spectra of a series of related Group IVa compounds. J. Phys. Chem..

[36] Wu T., Wang Y., Li X., Wu Y., Meng X., Cui D., Yang X., Han L. (2019). Efficient Defect Passivation for Perovskite Solar Cells by Controlling the Electron Density Distribution of Donor-π-Acceptor Molecules. Adv. Energy Mater..

[37] Jung H., Choi H., Kim S., Lee H., Kim Y., Yu J. (2017). The influence of N-doping types for carbon nanotube reinforced epoxy composites: A combined experimental study and molecular dynamics simulation. Compos. Part A Appl. Sci. Manuf..

[38] Jiang R., Liu N., Gao S., Mamat X., Su Y., Wagberg T., Li Y., Hu X., Hu G. (2018). A Facile Electrochemical Sensor Based on PyTS–CNTs for Simultaneous Determination of Cadmium and Lead Ions. Sensors.

[39] Chen H., Yu F., Wang G.G., Chen L., Dai B., Peng S. (2018). Nitrogen and Sulfur Self-Doped Activated Carbon Directly Derived from Elm Flower for High-Performance Supercapacitors. ACS Omega.

[40] Deng H., Yao L., Huang Q., Su Q., Zhang J., Zhang F., Du G. (2017). Facile assembly of a S@carbon nanotubes/ polyaniline/graphene composite for lithium–sulfur batteries. RSC Adv..

[41] Xu X., Zhang H., Li E., Ru P., Chen H., Chen Z., Wu Y., Tiana H., Zhu W. (2020). Electron-enriched thione enables strong Pb–S interaction for stabilizing high quality CsPbI3 perovskite films with low-temperature processing. Chem. Sci..

