# Supplementary material for: Polymer Backbone Stabilized Methylammonium Lead Bromide Perovskite Nano Islands
Source: Nanomaterials (Basel). 2023 Oct 12;13(20):2750. doi: 10.3390/nano13202750 (PMC10609000; doi:10.3390/nano13202750)
Supplement: Supplementary file 1 [file nanomaterials-13-02750-s001.zip › nanomaterials-2631661-supplementary.pdf]

nanomaterials

MDPI

Supporting Information

Polymer Backbone Stabilized Methylammonium Lead Bromide Perovskite Nano Islands

Chinna Bathula [1], Soniya Naik [2], Atanu Jana [3], Ramasubba Reddy Palem [4], Aditya Narayan Singh [5], Mohammad Rafe Hatshan [6], Suresh D. Mane [7] and Hyun-Seok Kim [1,*]

[1] Division of Electronics and Electrical Engineering, Dongguk University-Seoul, Seoul 04620, Republic of Korea; cdbathula@dongguk.edu
[2] Chemical and Materials Engineering Department, University of Alberta, Edmonton, AB T6G 2H5, Canada; soniya.d.naik1990@gmail.com
[3] Division of Physics and Semiconductor Science, Dongguk University, Seoul 04620, Republic of Korea; atanujanaic@gmail.com
[4] Department of Medical Biotechnology, Dongguk University, 32 Dongguk-ro, Ilsandong-gu, Goyang 10326, Republic of Korea; palemsubbareddy@gmail.com
[5] Department of Energy and Materials Engineering, Dongguk University-Seoul, Seoul 04620, Republic of Korea; aditya@dongguk.edu
[6] Department of Chemistry, College of Science, King Saud University, P.O. Box 2455, Riyadh 11451, Saudi Arabia; mhatshan@ksu.edu.sa
[7] D.Y. Patil Pratisthan's College of Engineering, Salokhe Nagar, Kolhapur 416007, Maharashtra, India; mane.suresh@gmail.com
* Correspondence: hyunseokk@dongguk.edu

Index

SI. No.	Content	Page No.
**Figure S1.**	FTIR spectra for PDT, MNI-PDT and MNI.	**2**
**Figure S2.**	XPS survey spectra of MNI-PDT composite.	**2**

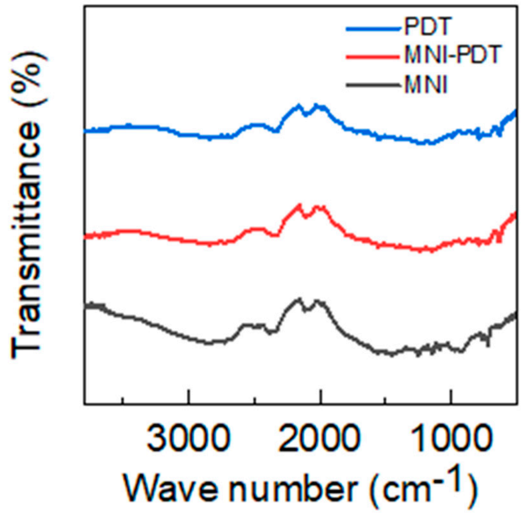

Figure S1. FTIR spectra for PDT, MNI-PDT and MNI.

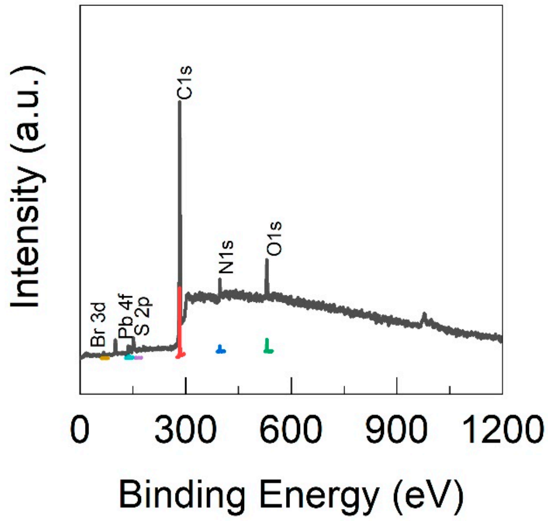

Figure S2. XPS survey spectra for MNI-PDT
